# Real‐life application of respiratory oscillometry in pediatric asthma outpatient care: Feasibility and methodological aspects

**DOI:** 10.14814/phy2.70735

**Published:** 2026-02-06

**Authors:** Charlotte Heijkenskjöld Rentzhog, Andrei Malinovschi, Kjell Alving

**Affiliations:** ^1^ Department of Women's and Children's Health, Pediatrics Uppsala University Uppsala Sweden; ^2^ Department of Medical Sciences, Clinical Physiology Uppsala University Uppsala Sweden

**Keywords:** asthma, clinical performance, feasibility, forced oscillation technique, pediatric, respiratory oscillometry

## Abstract

Asthma diagnosis can be challenging in children. Spirometry is highly effort‐dependent and often normal in early disease. Forced oscillation technique (FOT) is an alternative to spirometry and is performed during tidal breathing. We investigated the feasibility of FOT with fewer acquisitions than suggested by technical standards in a pediatric outpatient care setting. We also studied the influence of tidal breathing patterns on FOT indices. Finally, the clinical utility of FOT was compared with spirometry. Ninety‐five children aged 3–12 years performed FOT with single‐frequency mode of 8 Hz (Resmon Pro, ResTech, Italy) during initial asthma assessment or follow‐up. School‐aged children (*n* = 61) also performed spirometry. In preschool age (<6 years), 94% managed one and 74% managed two approved FOT acquisitions, whereas in school‐age 100% and 92% managed correspondingly. Reasonable agreement between two device‐approved FOT measurements was found. No difference in FOT values was found with spontaneous higher respiratory rates or tidal volumes. Bronchodilation responses measured with FOT, but not spirometry, were associated with ongoing anti‐inflammatory asthma medication. FOT with an integrated quality control was highly feasible in children 3–12 years. Reasonable agreement between two device‐approved acquisitions was found suggesting one measurement might suffice. Deviations from normal tidal breathing had little influence on FOT results.

## 1 INTRODUCTION

Asthma is a chronic disease with variable airway inflammation that comprises multiple phenotypes, and a clear‐cut diagnosis can be challenging, especially in younger children. Dynamic spirometry requires maximal respiratory efforts, is difficult for preschool children to perform, and is often normal despite a clinical picture of asthma in early disease (Yang et al., 2019). Respiratory oscillometry offers a complementary and sensitive measurement that is easier to perform since it only requires tidal breathing.

In clinically applicable respiratory input oscillometry, a low‐amplitude pressure signal is applied to the airways during tidal breathing through a mouthpiece while a pneumotachograph measures pressure and flow changes which allow calculation of airway impedance—the overall resistive and reactive forces to be overcome for an oscillating signal passing through the respiratory system. The impedance is by mathematical modeling further divided into variables of respiratory system resistance and reactance (Bosse, 2022).

Common commercial devices use either impulse oscillometry (IOS) or forced oscillation technique (FOT) signal systems, with signal frequencies between 4 and 40 Hz. Outcome impedance values are mean values from multiple time points collected during one acquisition consisting of a series of consecutive uninterrupted tidal breaths. Some devices also present mean inspiratory and expiratory impedance measures. Respiratory impedance varies over a normal breath cycle and inherently, mean variables present higher variability compared to spirometry (Sol et al., 2019).

Impedance variables acquired by oscillometry reflect the overall airways, lung tissue, and chest wall impedance. In airway obstructive disease and provocation tests, raised respiratory resistance as well as more negative values of respiratory reactance, compared to normal values for age and chosen input frequency in the range 4–40 Hz, are found (Oostveen et al., 2003; Wesseling et al., 1993). For valid measurements, control over body position, tongue positioning, cheek stabilization, coughing, leakage at the mouthpiece, and use of a nose‐clip is necessary. Some devices offer inbuilt algorithms that exclude whole breath cycles with nonphysiological output data. Technical standards published by the European Respiratory Society (ERS) (King et al., 2020) recommend at least three accepted breath cycles for one acquisition, and preferably three acquisitions to assess repeatability. The coefficient of variation for the mean resistance value between repeated acquisitions is suggested to be maximally 10% in adults and 15% in children.

For preschool children the task to remain focused and cooperative during multiple repeated acquisitions is challenging. There is limited published data on how duration and number of acquisitions affect quality of variables in respiratory oscillometry. To the best of our knowledge, no data on the influence of respiratory rate and tidal volume beyond normal tidal breathing at rest in preschool children was presented. The primary aim of our study was to establish the overall feasibility of respiratory oscillometry acquired by FOT with single‐frequency mode of 8 Hz with an integrated control algorithm in children 3–12 years of age in a real‐life pediatric outpatient setting. Specifically, the influence of the number of approved breaths in acquisition, and the respiratory rate and tidal volume performance on oscillometric indices were studied. Furthermore, clinical utility of FOT measurement in asthma assessment was examined and compared with spirometry.

## 2 MATERIALS AND METHODS

### 2.1 Subjects and clinical asthma assessment

From a total of 112 children, aged 3–12 years, invited through their caregivers to participate during a planned visit for follow‐up or assessment of suspected asthma at a secondary‐care pediatric clinic in Uppsala, Sweden, between January 2019 and October 2021, 95 children were included, whereof 91 were clinically diagnosed as asthma. Fourteen children/caregivers declined to participate, and three were excluded due to prematurity <32 weeks, or pseudo‐croup. Subjects' age, sex, height and weight, and information relevant for asthma assessment was collected through a questionnaire based partly on a standardized international allergy/asthma questionnaire (ISAAC) (Asher et al., 1995), and partly on a previous study questionnaire (MIDAS) (Heijkenskjold‐Rentzhog et al., 2014; Patelis et al., 2014). Asthma Control Test (ACT) according to age was used to assess subjects'/caregivers' perspective of symptom control, with ACT score <20 implying unsatisfactory symptom control (Liu et al., 2010; Nathan et al., 2004).

### 2.2 Blood analysis for aeroallergen sensitization assessment

Blood samples were collected for measurement of IgE antibodies to important airborne allergens: cat, dog, horse, mite, birch, timothy, mugwort, and mold (Phadiatop; Thermo Fisher Scientific, Uppsala, Sweden). Aeroallergen sensitization was defined as having IgE antibodies against Phadiatop ≥0.35 kU/L.

### 2.3 Lung function assessment

FOT measurements were performed with a Resmon PRO FULL (ResTech, Milano, Italy) device according to the manufacturer's recommendations with the use of single‐frequency mode of 8 Hz, suggested for children, and before spirometry. The children were instructed to place teeth and lips around the mouthpiece, close lips and keep a near‐normal breathing pattern during the procedure while comfortably placed in the sitting position on a chair or in the caregiver's lap, with the head slightly tilted upwards and cheeks held by the nurse or caregiver. A disposable mouthpiece with an anti‐bacterial filter and a nose‐clip were used. Before start of data collection during the first few breaths, wave amplitude was automatically adjusted for subject data, followed by a registration period. An approved measurement was defined by at least one acquisition with at least five algorithm‐approved full breath cycles. An internal algorithm automatically excluded whole breaths with data implying nonphysiological conditions from leakage, coughing, or tongue malposition. An experienced nurse performed the test session with control over the child's breath maneuver in the room. No further evaluation of the test results was performed during the test session. Two acquisitions were attempted both pre‐ and post‐bronchodilation. During acquisition, 200 data points per second are gathered, and mean impedance values are internally calculated. Impedance values provided were inspiratory, expiratory, and total breath (inspiratory and expiratory) resistance (R) and reactance (X). Variability within‐ and between repeated acquisitions were calculated afterwards during data analysis and not used for the original acquisition approval. Difference between inspiratory and expiratory phase impedance was calculated in magnitude and proportion and presented as Delta‐R, Delta‐X and Delta‐R%, Delta‐X%, respectively. A bronchodilator response test was performed with salbutamol (spray and spacer). Subjects 3–5 years were given 200 micrograms and from 6 years 400 micrograms. Post‐bronchodilation measurements were performed after 15 min. Flow‐volume curves were obtained in accordance with the American Thoracic Society (Miller et al., 2005) using a desktop spirometer (VyntusSpiro, Vyaire, Chicago, USA) in children aged 6 years and above. Bronchodilator response was expressed as percentage change from the initial value for both spirometry and oscillometry. A positive bronchodilator response was defined for spirometry according to 2005 ATS/ERS guidelines (Miller et al., 2005) as ≥12% increase in FEV_1_ or FVC, and for oscillometry as ≥40% reduction in resistance or ≥50% increase (i.e., change towards less negative value) in reactance (King et al., 2020). Impedance values at baseline were also presented as upper‐ and lower‐limit of normal according to Ducharme et al. (2022).

### 2.4 Ethics

Written informed consent for participation in the study was obtained from the legal guardians. The study was approved by Uppsala Regional Ethics Review Board (approval number 2018/257) and conducted in accord with the Declaration of Helsinki.

### 2.5 Statistical analysis

Statistical analyses were performed with STATA/IC 12.1 (StataCorp LP, College Station, TX, USA). GraphPad Prism 10.4.0 was used for graphical presentations. Linear regression models were used for analyses between (log‐transformed) resistance estimates. Nonparametric tests were used for correlation analysis between reactance estimates and unadjusted impedance variables in dichotomized subject groups, and bronchodilation response in relation to asthma characteristics. Coefficient of variation (SD/mean × 100) was used to present variability within‐ and between repeated acquisitions. Agreement between two acquisitions was presented using Bland–Altman plots, with Pitman's test of difference. A *p* value of <0.05 was considered significant.

## 3 RESULTS

Characteristics of 95 children, mean age 7.4 years, of which 36% were preschoolers and 65% of male sex, are presented in Table 1.

**TABLE 1:** Asthma characteristics of the study group (*n* = 95) of children age 3–12 years attending for planned asthma assessment.

	Preschool age	School age
	(3–5 years)	(6–12 years)
	(*N* = 34)	(*N* = 61)
Aeroallergen sensitization	24%	54%
ACT score^a^	21.3 +/− 3.6	21.0 +/− 3.9
Any asthma medication last 12 months *n* (%)^b^	26 (81)	52 (85)
Ongoing anti‐inflammatory asthma medication last 3 weeks *n* (%)	10 (30)	22 (36)
Asthma medication ≥4 months last 12 months *n* (%)	6 (18)	16 (26)
Emergency visit obstructive episode ever *n* (%)^c^	13 (41)	33 (57)
Emergency visit last 12 months *n* (%)	11 (33)	16 (26)
Pre‐BD FEV_1_ (% predicted)^d^		92.4 (82.6–102.1)
Pre‐BD FEV_1_ < 80% predicted *n* (%)^d^		9 (15)
Pre‐BD FEV_1_/FVC		0.86 (0.79–0.89)
FEV_1_ BD response ≥ 12% *n* (%)		8 (13)
FVC BD response ≥ 12%		0
Pre‐BD R_8_ (cm H_2_O/L/s)	10.3 (8.8–12.0)	7.1 (5.7–8.5)
Pre‐BD R_8_ ≥ Ducharme ULN *n* (%)^e^	11 (34.4)	17 (27.9)
Pre‐BD X_8_ (cm H_2_O/L/s)	−3.1 (−4.3, −2.0)	−1.5 (−2.2, −1.1)
Pre‐BD X_8_ ≤ Ducharme LLN *n* (%)^e^	9 (28.1)	6 (9.8)
Pre‐DeltaR cmH_2_O/L/s	1.5 (0.67–2.45)	1.2 (0.66–1.88)
Pre‐DeltaR% of Rtotal	15.8 (5.7–27.3)	16.6 (10.8–23.7)
Pre‐DeltaX cmH_2_O/L/s	0.6 (0.07–1.47)	0.5 (0.2–1.1)
Pre‐DeltaX% of X total	21.8 (2.6–37.8)	42.4 (17.9–72.7)
Post‐BD R_8_ (cm H_2_O/L/s)	8.3 (7.2–9.1)	5.3 (4.4–6.2)
Post‐BD X_8_ (cm H_2_O/L/s)	−2.0 (−2.6, −1.5)	−0.9 (−1.1, −0.6)
R_8_ BD response ≥ 40% *n* (%)	0	9 (15)
X_8_ BD response ≥ 50% *n* (%)	8 (27)	21 (34)

*Note*: B‐count and ACT‐score presented as mean value with standard deviation. Lung function parameters and FeNO presented as median value with interquartile range (IQR) Impedance values presented from first accepted acquisition.

Abbreviations: ACT, asthma control test; BD, bronchodilation; Delta‐R, difference in R (expiratory) and R (inspiratory), R(exp)‐R(insp); Delta‐R(%), Delta‐R/R_8_to × 100 and for Delta‐X correspondingly; FEV_1_, forced expiratory volume at 1 s; FVC, forced vital capacity; R_8_, FOT resistance at 8 Hz; X_8_, FOT reactance at 8 Hz.

^a^ Data available in *n* = 31 and 60, respectively.

^b^ Data available in *n* = 32 preschooler.

^c^ Data available in *n* = 32 and 58, respectively.

^d^ Solymar reference equation (Solymar et al., 1980).

^e^ ULN (upper limit of normal 1.645 SD), LLN (lower limit of normal ‐1.645 SD).

### 3.1 Feasibility of FOT measurement

In preschool age, 94% and 88% accomplished at least one approved pre‐ and post‐bronchodilation acquisition, while two approved acquisitions were accomplished in 74% and 56%, respectively (Table 2a,b). All school‐age children accomplished at least one approved acquisition pre‐ and post‐bronchodilation, and two acquisitions were achieved in 92% and 89%, respectively. No difference in age within subgroup, sex, ACT score, or ongoing asthma medication was found between children with one or two approved acquisitions.

**TABLE 2:** Proportion of all preschool‐ and school‐age children that accomplished (a, b): At least one, and repeated, algorithm approved acquisitions and (c, d): At least one, and repeated, algorithm approved acquisitions with the post hoc added requirement of variability‐within‐acquisition ≤15% for FOT 8 Hz measurement.

	All subjects	Age 3–5 years	Age 6–12 years
	(*N* = 95)	(*N* = 34)	(*N* = 61)
All approved acquisitions			
a/Pre bronchodilation			
One approved acquisition (*n* (%))^a^	93 (98)	32 (94)	61 (100)
Two approved acquisitions (*n* (%))^a^	81 (85)	25 (74)	56 (92)
b/Post‐bronchodilation			
One approved acquisition (*n* (%))^a^	91 (96)	30 (88)	61 (100)
Two approved acquisitions (*n* (%))^a^	73 (77)	19 (56)	54 (89)
Acquisitions with ≤15% variability‐within‐acquisition			
c/Pre bronchodilation			
One approved acquisition (*n* (%))^b^	80 (84)	23 (68)	57 (93)
Two approved acquisitions (*n* (%))^b^	65 (68)	17 (50)	48 (79)
d/Post‐bronchodilation			
One approved acquisition (*n* (%))^b^	70 (74)	24 (71)	46 (75)
Two approved acquisitions (*n* (%))^b^	46 (48)	11 (32)	35 (57)

^a^ Algorithm‐approved acquisition: at least five algorithm‐approved full breaths.

^b^ At least five algorithm‐approved full breaths, and ≤15% variability‐within‐acquisition in post hoc calculation.

In Table 2c,d approved acquisitions are presented with post hoc use of a stricter definition and requirement of variability ≤15% in total resistance measures within acquisition.

### 3.2 Variability and reliability of FOT impedance values

**FIGURE 1:**
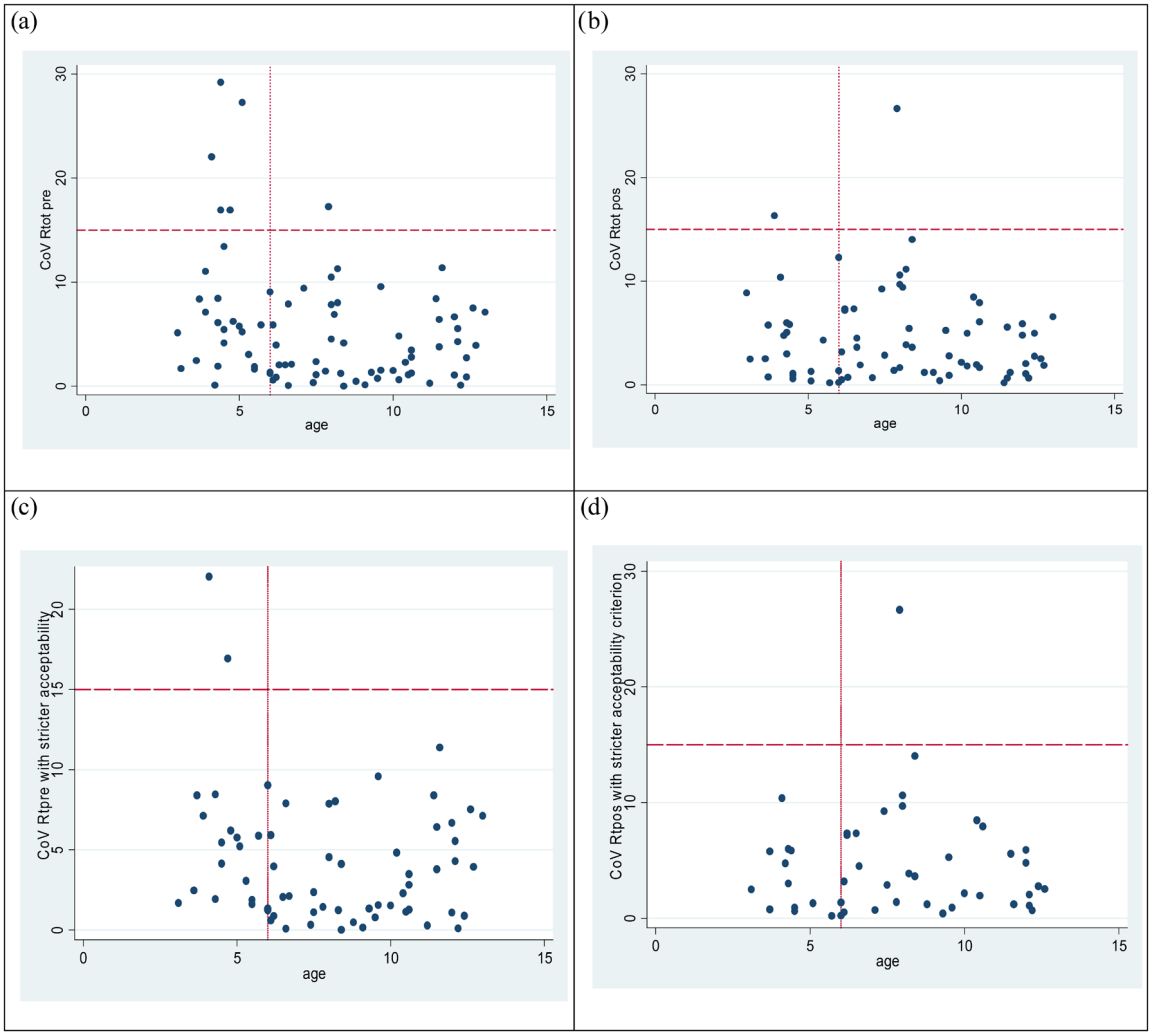
(a–d) Scatter plot according to age for coefficient of variation (CoV %) of duplicate acquisitions of FOT 8 Hz resistance (a) pre‐ and (b) post‐ bronchodilation measurements, and (c) pre‐ and (d) post‐ bronchodilation measurements for only acquisitions with variability‐within‐acquisition ≤15%. Dashed lines indicate CoV 15% and dotted lines delineate preschool‐ and school‐age children, respectively. (a) CoV of duplicate algorithm‐approved acquisitions for resistance at 8 Hz pre‐bronchodilation. (b) CoV of duplicate algorithm‐approved acquisitions for resistance at 8 Hz post‐bronchodilation. (c) CoV of duplicate algorithm‐approved acquisitions with variability‐within‐acquisition ≤15% for resistance at 8 Hz pre‐bronchodilation. (d) CoV of duplicate algorithm‐approved acquisitions with variability‐within‐acquisition ≤15% for reactance at 8 Hz post‐bronchodilation.

For total resistance measures the coefficient of variation between duplicate acquisitions (CoV) was in preschool age 5.9% (3.1–11.0 IQR) and in school age 2.5% (1.1–6.8 IQR). CoV ≤15% was found in 20/25 of preschool and in 55/56 of school age (Figure 1).

With regard to variability‐within‐acquisition for total resistance and first accepted measurement, 72% (23/32) of preschoolers presented <15% (Table 2c) and 93% (57/61) correspondingly in school age. With post hoc exclusion based on variability‐within‐acquisition <15%, only a few duplicate measurements with CoV >10% were found all ages (Figure 1).

For total reactance measures the CoV was in preschoolers 12.9% (7.4–22.6 IQR) and in school‐age 8.5% (4.5–14.8 IQR) and variability‐within‐acquisition in first accepted measurement correspondingly 29.0% (20.3–52 IQR) and 30.1% (18.3–41.2).

**FIGURE 2:**
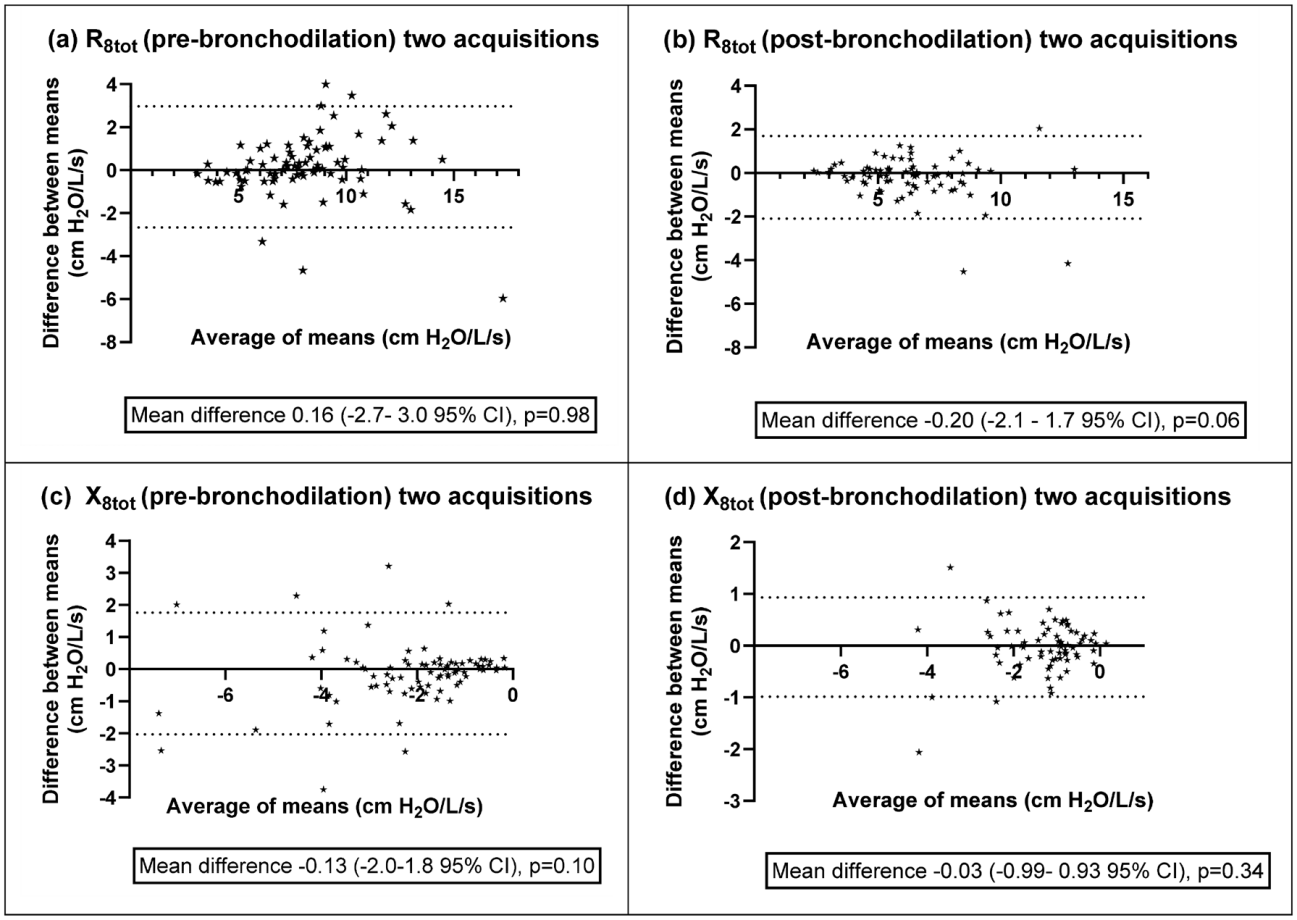
(a–d) Bland–Altman plots presenting the difference between means of two‐ over the average of means of two‐ acquisitions, for total (a) pre‐ and (b) post‐ bronchodilation resistance (c) pre‐ and (d) post‐ bronchodilation reactance at 8 Hz with dotted lines representing Limit of Agreement at 95% CI and with Pitman's test of difference in variance *p* value.

Agreement between two approved acquisitions was reasonable (Figure 2) for impedance values presented as Bland–Altman plots, with no significant correlation between the differences and the means. All further analyses are performed with first algorithm‐approved acquisition according to Table 2a,b, if not otherwise specified.

### 3.3 Number of approved breaths in acquisition and FOT impedance measures

No difference in impedance values was found between acquisitions with five or more algorithm‐approved breaths (Table 3). Age, height, sex, and asthma characteristics were no different between groups.

**TABLE 3:** Subgroups of children according to the number of approved breaths in first approved acquisition.

Number of approved breaths in an acquisition	5	6–9	10	*p* Value (Kruskal Wallis)
	(*N* = 37)	(*N* = 44)	(*N* = 12)	
Age (years)	7.0+/−2.8	7.8 +/−2.8	8.4+/−3.0	0.27
Height (cm)	117.7 +/− 18.5	126.5 +/−17.7	136 +/− 21.8	0.20
ACT, median	22	21	23	0.30
R_8_ tot (cm H_2_O/Lxs)	8.9 (7.4–9.8)	7.3 (5.9–8.9)	8.2 (5.9–10.1)	0.19
Delta‐R (cm H_2_O/Lxs)	1.5 (2.3–0.8)	1.0 (1.8–0.4)	1.2 (1.7–0.8)	0.39
Delta‐R (%)	18.0 (10.7–24.9)	16.2 (7.9–22.6)	15.5 (13.0–20.7)	0.78
Within‐acquisition variability of R (%)	10.0 (6.8–11.9)	10.3 (8.4–12.6)	9.8 (8.9–10.1)	0.34
X_8_ tot (cm H_2_O/Lxs)	−2.1 (−1.4, −2.9)	−1.6 (−1.1, −3.1)	−1.9 (−0.9, −3.3)	0.41
Delta‐X (cm H_2_O/Lxs)	−0.6 (−0.1, −1.6)	−0.5 (−0.2, −1.0)	−0.8 (−0.3, −1.7)	0.6
Delta‐X (%)	30.8 (5.4–60.7)	36.3 (30.2–65.0)	47.4 (20.9–72.9)	0.5
Within‐acquisition variability of X (%)	26.9 (18.6–59.7)	32.1 (20.0–45.3)	26.3 (20.1–32.5)	0.86
Age 3–5 years (*n* (%))	16 (43)	13 (30)	3 (25)	0.33

*Note*: All FOT values are median (IQR) if not otherwise indicated.

Abbreviations: Delta‐R, difference in R (expiratory) and R (inspiratory), R(exp)‐R(insp); Delta‐R(%), Delta‐R/R_8_tot × 100 and for Delta‐X correspondingly; R_8_, resistance at 8 Hz; X_8_, reactance at 8 Hz.

### 3.4 The influence of respiratory rate and tidal volume on FOT resistance measures

In multiple regression models adjusted for height, neither respiratory rate nor tidal volume as percentage difference from predicted did influence absolute resistance values (*p* = 0.29 and *p* = 0.20, respectively). Results remained with analyzes of dichotomized increase in respiratory rate (above 99th percentile according to Fleming et al. (2011)) or tidal volume (>150% of predicted value, defined as 10 mL/kg) within the borders of acceptance by the algorithm (*p* = 0.19 and *p* = 0.08, respectively).

### 3.5 Bronchodilator response in spirometry and FOT measurements in relation to asthma characteristics

**FIGURE 3:**
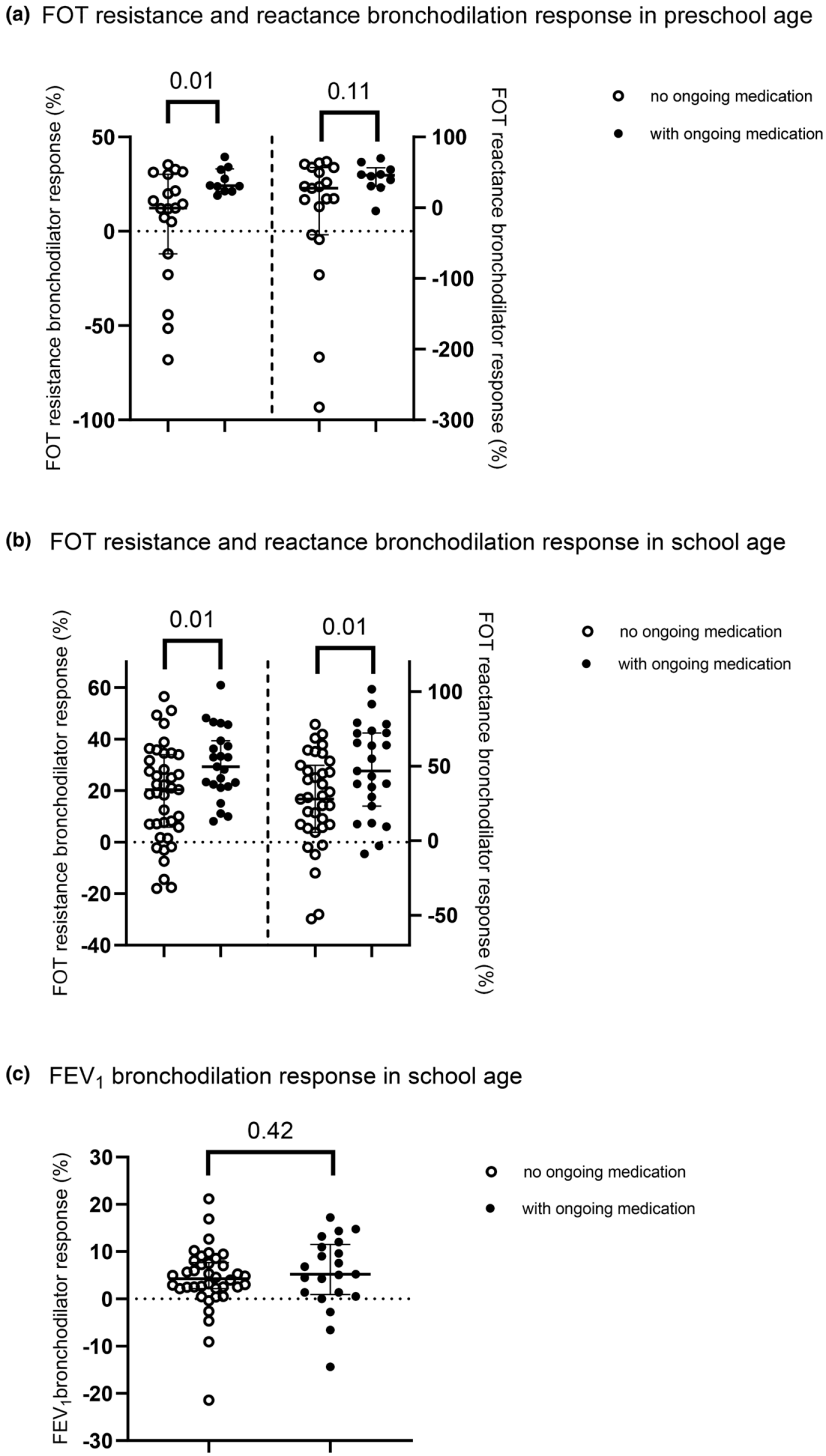
(a–c) Box plot presenting bronchodilator response (%) in: (a) preschool‐age children FOT resistance numeral decrease (left) and reactance numeral increase (right), (b) school age children for FOT resistance numeral decrease (left) and reactance numeral increase (right), and (c) school age children for FEV_1_; in subgroups with or without ongoing anti‐inflammatory asthma medication during last 3 weeks. Bronchodilator responses indicated for subgroups are median values and interquartile range.

In school‐age children, no association was found between spirometry bronchodilation response and ongoing anti‐inflammatory asthma medication (see Figure 3c), duration of treatment last year, asthma control (ACT score), emergency visits, or aeroallergen sensitization.

FOT resistance bronchodilation response was positively associated with ongoing asthma medication (−26.3% (21.4–36.7 IQR) versus −18.5% (5.1–31.3 IQR) decrease in resistance), *p* < 0.001, and aeroallergen sensitization (−25.6% (19.1–35.8 IQR) versus −20.5% (8.1–30.2 IQR)), *p* = 0.03. Also, FOT reactance bronchodilator response associated with ongoing medication (46.9% (28.9–67.7 IQR) versus 27.7% (1.4–50.5 IQR)) change in reactance towards less negative values, *p* = 0.003 and aeroallergen sensitization (45.0% (23.7–68.8 IQR) versus 28.5% (1.4–47.3 IQR)), *p* = 0.004 for all children. The association remained for resistance and ongoing medication when analyzing school age‐ and preschool age‐ groups separately (*p* = 0.02 and *p* = 0.02, respectively), (Figure 3a,b), and as for reactance only in school‐age with ongoing asthma medication and aeroallergen sensitization (*p* = 0.01 both).

### 3.6 FOT resistance difference in expiration versus inspiration, upper limit of normal and association with asthma characteristics

A positive association between Delta‐R and ongoing medication last 3 weeks (1.8 cmH_2_O/L/s (0.8–2.4 IQR) vs. 1.1 cmH_2_O/L/s (0.5–1.8 IQR) *p* = 0.03) was found, but no difference was found regarding DeltaR%. Also, for Delta‐X an association was found (0.9 cmH_2_O/L/s (0.4–1.8 IQR) vs. 0.4 cmH_2_O/L/s (0.1–0.9 IQR), *p* = 0.03), but not for Delta‐X%. Additionally, when applying the more recently presented reference equations (Ducharme et al., 2022), an association between resistance values above upper limit of normal (ULN: 1.645 standard deviations) and ongoing medication, but no other asthma characteristics, was found (*p* = 0.02). No association was found with reactance below lower limit of normal (LLN) and any asthma characteristics.

## 4 DISCUSSION

The main findings in the present study were a reasonable agreement between two acquisitions for FOT with single‐frequency mode of 8 Hz resistance despite no visual inspection of data during acquisition, and that deviations in normal tidal breathing at rest within acceptance of the internal algorithm had little influence on FOT resistance values. We found that 94% of pre‐school children managed one algorithm‐approved acquisition, and 74% managed two acquisitions, whereas FOT measurement feasibility was excellent in school‐aged children. No differences in impedance values were found with longer acquisition times compared to five approved breath cycles. Bronchodilation responses measured with FOT, but not spirometry, were associated with ongoing asthma medication indicating symptomatic disease.

Technical standards (King et al., 2020) suggest at least three repeated acquisitions both pre‐ and post‐ bronchodilation. This may limit the clinical use in the preschool age. Ducharme et al. (2022) presented in healthy children 3–17 years of age an overall excellent feasibility when post hoc two technically accepted acquisitions with a coefficient of variation of ≤10% were decided enough. Although, in their material, in age 3–4 years, four out of 16 children did not manage two accepted acquisitions. In the present clinical study, two acquisitions with internal algorithm‐based quality control, pre‐ and post‐ bronchodilation, were aimed for. In school‐age a majority managed two acquisitions both pre‐ and post‐ bronchodilation. In pre‐school age, although a high proportion managed one pre‐ and post‐bronchodilation acquisition, only 74% and 56% respectively managed two, mostly due to lack of focus and willingness to maintain posture, lip seal and/or nose clip.

Calogero et al. (2010) presented in healthy children 2.9–6.1 years of age CoV from 3 to 5 acquisitions with FOT resistance at 8 Hz to be 5.9% and Knihtila et al. (2017) presented excellent repeatability for triplicate measurements at 5 Hz in children with the use of IOS. In the present study, we found a CoV between duplicate acquisitions for resistance at 8 Hz in preschool‐ and school‐age to be 5.9% and 2.5%. Our study is a real‐life set up representing conditions for asthma assessment in a secondary‐care pediatric clinic. No difference in patient characteristics was found between children that succeeded with two or only one acquisition. Whenever possible, technical standard recommendations of triplicate acquisitions remain standard for quality assurance of test results. However, our results support that less than three acquisitions may be sufficient, especially with an internal algorithm in the device to exclude nonphysiological measures and with a trained person in control of the child and the test situation.

With regard to within‐acquisition variability, calculated post hoc in our study, disease‐related higher variability cannot be ruled out, highlighted by the association between DeltaR and ongoing anti‐inflammatory medication. The within‐acquisition variability of resistance found to be >15% for some baseline accepted measurements could have been minimized with a visual inspection of output measurements during test sessions. In the present study, the quality check was performed by a device‐specific automated algorithm that excludes whole breaths with nonphysiological flow and impedance registrations with no visual inspection of the output measures during test session. We believe that a graphic presentation of output data with real‐time measures of tidal volume, flow, and within‐acquisition variability of resistance, now possible with updated software, may facilitate exclusion of whole breaths collected with algorithm‐accepted disturbances during data sampling. More studies on children with asthma and healthy controls are warranted to establish the minimal criteria needed for FOT measurements with adequate quality.

No difference in impedance values or variability was found between acquisitions over 5, 6–9, or 10 approved breath cycles. Thus, five approved breaths seemed to be sufficient in children 3–12 years of age. Variations in respiratory rate and tidal breathing volumes were found in our participants, despite instructions to perform regular tidal breathing. However, neither raised respiratory rate nor higher tidal volume compared to reference values was associated with altered impedance values when adjusting for patient height. Previous findings in adults presented an effect of voluntarily raised respiratory rates, in the range of two to three times higher than the normal rate, with altered impedance values, especially in symptomatic individuals (Oppenheimer et al., 2009). Studies in children from age 6 years performing exercise lung function tests found no association between breathing pattern parameters and impedance values shortly after exercise (Barreto et al., 2023; Veneroni et al., 2022). In our material, we compared measurements with spontaneously raised respiratory rates above the 99th percentile of the age range and tidal volumes above 150% of the calculated reference values. The importance of our finding is that spontaneously and moderately raised respiratory rates and/or tidal volumes in children with diagnosed or suspected asthma had no effect on height‐adjusted impedance values.

The feasibility of spirometry in early school‐age children with suspected asthma is limited to some extent (Onisor & Turner, 2023). Beside the cases in whom spirometry cannot be reliably performed, a normal spirometry result is fairly common in early stages of disease. The evidence of the clinical use of respiratory oscillometry complementary to standard diagnostics is mounting, especially in childhood asthma (Elenius et al., 2021; Galant et al., 2017; Kaminsky et al., 2022; Vielkind et al., 2022; Xepapadaki et al., 2023). However, the most useful way to define a bronchodilator response in the clinic needs to be determined. In the present study, we found an association between the bronchodilation response by FOT and ongoing anti‐inflammatory asthma medication, whereas no association was found between any spirometry variable and asthma characteristics. Interestingly, an association with ACT score was not found, which might imply difficulties in discriminating perceived airway symptoms caused by upper airway pathology, viral respiratory tract infections, and anxiety, to mention some other causes of symptoms in a common pediatric setting. Studies in both childhood and adult asthma have suggested that self‐reported asthma control and lung function may represent different domains of asthma (Green et al., 2013; Park et al., 2015).

A limitation of the study may be the lack of bronchodilation response data in healthy control subjects. Previously, at least a 35%–40% reduction in resistance has been suggested as thresholds in different pediatric populations (King et al., 2020). In our study, only a few children presented a bronchodilation response in FOT resistance above the most commonly recommended threshold of 40%. On the other hand, a similar proportion of individuals exhibited a significant response in FEV_1_, which probably highlights the generally low sensitivity of the bronchodilation test. Our study set up implies a limitation with regard to inclusion of children of different pediatric asthma phenotypes, with or without ongoing asthma treatment plans. However, this was also a strength since it allowed for the study of feasibility and factors affecting FOT measurement in a real‐life clinical setting. Reactance measures in our data presented an overall higher variability than resistance measures both within‐ and between acquisitions, constituting a challenge in interpretation and significance with regard to bronchodilator response, especially without healthy control data. With the application of newly presented reference equations and ULN/LLN for impedance measures to our data, the association between reactance measurements and ongoing medication was no longer found.

In conclusion, FOT is feasible even in young children with asthma where spirometry cannot be performed and one accepted FOT measurement for pre‐ and post‐ bronchodilation assessment seems to be sufficient for clinical information. Furthermore, five accepted respiratory cycles during measurement seem enough and minor variations in respiratory pattern during measurement do not significantly change FOT outcome variables. Our results suggest that FOT bronchodilation response is a sensitive objective measure of ongoing variable airway obstructive pathology in preschool children, and in school‐age children where spirometry presents a normal outcome. Further pediatric studies including also healthy controls for the estimation of optimal cutoffs to define a clinically significant bronchodilator response by FOT measurement are warranted.

## AUTHOR CONTRIBUTIONS

CHR, AM, and KA contributed substantially to the conception and design of the work. CHR and KA contributed to data collection and CHR performed the patient management. CHR and AM contributed to the statistical analysis and CHR performed the manuscript drafting. AM, KA, and CHR contributed substantially to the interpretation of results, literature review, critical revision, and final approval of the manuscript. All authors read and approved the final version of the manuscript.

## FUNDING INFORMATION

This study was supported by the Swedish Asthma and Allergy Association's Research Foundation.

## CONFLICT OF INTEREST STATEMENT

The authors declare no conflicts of interest.

## ETHICS STATEMENT

The study was approved by Uppsala Regional Ethics Review Board (approval number 2018/257).

## Data Availability

Data cannot be made freely available as they are subject to secrecy in accordance with the Swedish Public Access to Information and Secrecy Act. Data can be made available to researchers upon request, after approval from the Swedish Ethical Review Authority has been obtained.
